# CLEC10A Is a Specific Marker for Human CD1c^+^ Dendritic Cells and Enhances Their Toll-Like Receptor 7/8-Induced Cytokine Secretion

**DOI:** 10.3389/fimmu.2018.00744

**Published:** 2018-04-27

**Authors:** Lukas Heger, Silke Balk, Jennifer J. Lühr, Gordon F. Heidkamp, Christian H. K. Lehmann, Lukas Hatscher, Ariawan Purbojo, Arndt Hartmann, Fayna Garcia-Martin, Shin-Ichiro Nishimura, Robert Cesnjevar, Falk Nimmerjahn, Diana Dudziak

**Affiliations:** ^1^Department of Dermatology, Laboratory of Dendritic Cell Biology, Friedrich-Alexander Universität Erlangen-Nürnberg (FAU), University Hospital Erlangen, Erlangen, Germany; ^2^Department of Pediatric Cardiac Surgery, Friedrich-Alexander Universität Erlangen-Nürnberg (FAU), University Hospital Erlangen, Erlangen, Germany; ^3^Department of Pathology, Friedrich-Alexander Universität Erlangen-Nürnberg (FAU), University Hospital Erlangen, Erlangen, Germany; ^4^Graduate School of Life Science and Faculty of Advanced Life Science, Hokkaido University, Sapporo, Japan; ^5^Department of Biology, Chair of Genetics, Friedrich-Alexander Universität Erlangen-Nürnberg (FAU), Erlangen, Germany

**Keywords:** dendritic cell subpopulations, CLEC10A, lineage marker, antigen targeting, cDC2, CD1c^+^ dendritic cells

## Abstract

Dendritic cells (DCs) are major players for the induction of immune responses. Apart from plasmacytoid DCs (pDCs), human DCs can be categorized into two types of conventional DCs: CD141^+^ DCs (cDC1) and CD1c^+^ DCs (cDC2). Defining uniquely expressed surface markers on human immune cells is not only important for the identification of DC subpopulations but also a prerequisite for harnessing the DC subset-specific potential in immunomodulatory approaches, such as antibody-mediated antigen targeting. Although others identified CLEC9A as a specific endocytic receptor for CD141^+^ DCs, such a receptor for CD1c^+^ DCs has not been discovered, yet. By performing transcriptomic and flow cytometric analyses on human DC subpopulations from different lymphohematopoietic tissues, we identified CLEC10A (CD301, macrophage galactose-type C-type lectin) as a specific marker for human CD1c^+^ DCs. We further demonstrate that CLEC10A rapidly internalizes into human CD1c^+^ DCs upon binding of a monoclonal antibody directed against CLEC10A. The binding of a CLEC10A-specific bivalent ligand (the MUC-1 peptide glycosylated with N-acetylgalactosamine) is limited to CD1c^+^ DCs and enhances the cytokine secretion (namely TNFα, IL-8, and IL-10) induced by TLR 7/8 stimulation. Thus, CLEC10A represents not only a candidate to better define CD1c^+^ DCs—due to its high endocytic potential—CLEC10A also exhibits an interesting candidate receptor for future antigen-targeting approaches.

## Introduction

Dendritic cells (DCs) are potent antigen-presenting cells capable of inducing and regulating adaptive immune responses (1). They continuously sample pathogenic and harmless antigens from their surroundings. This is followed by the migration into lymphoid organs, accompanied by antigen processing and the presentation of the uptaken antigens as peptide MHC complexes to T cells. Tolerogenic T cell reactions are induced if danger signals are missing. In contrast, additional danger signals (such as TLR-mediated signals) allow for the full maturation of DCs and thereby the induction of life-long immunity against invading pathogens (1, 2).

Dendritic cells can be distinguished into plasmacytoid DCs (pDCs) and conventional DCs. pDCs are known for their ability to secrete high amounts of type 1 interferon in response to viral infections and a lower T cell stimulatory capacity (3–5). In mice and man, two major conventional DC subpopulations have been described. Murine conventional DCs can be separated into CD8^+^CD11b^−^/CD103^+^CD11b^−^ DCs (also named cDC1) and CD8^−^CD11b^+^ DCs (also named cDC2) (6–8). The first excel in cross-presentation of antigens to cytotoxic CD8^+^ T cells as well as T_H_1 CD4^+^ T cell responses, whereas the latter induce preferentially T_H_2 and T_H_17 responses (9–14). According to their expression profiles, human conventional DCs can be distinguished into CD141^+^ and CD1c^+^ DCs. CD141^+^ DCs share the expression of BATF3, IRF8, and XCR1 with murine CD8^+^CD11b^−^/CD103^+^CD11b^−^ DCs, whereas CD1c^+^ DCs are homologous to murine CD8^−^CD11b^+^ DCs in regard to their expression of SIRPα and IRF4 (15–18).

Due to their unique ability to induce naïve T cell responses, DCs are suitable targets for immunotherapeutic approaches for the therapy of cancer as well as infectious diseases. One promising strategy is the *in vivo* delivery of antigens to DCs using antibodies directed against endocytic surface receptors (19). Hereby, it is possible to induce protective as well as therapeutic immune responses (19–27). In order to harness DCs for antigen-targeting approaches, it is necessary to identify endocytic receptors specifically expressed on DCs. One suitable subclass of such endocytic receptors are C-type lectin receptors (CLRs). In mice, the specific expression of the CLRs DEC205 and DCIR2 allowed for the distinct targeting of the conventional DC subsets, leading to CD8^+^ or CD4^+^ T cell responses, respectively (9, 20, 28). In humans, DEC205 and DCIR (a homolog of murine DCIR2) are not only expressed by one specific DC subset, thereby hindering the direct translation into the human system (15, 29–31). Recently, CLEC9A was identified as a uniquely expressed CLR on murine CD8^+^CD11b^−^/CD103^+^CD11b^−^ DCs and human CD141^+^ DCs (21, 22, 32–35). However, a potential targeting receptor specifically expressed on human CD1c^+^ DCs is still missing.

Transcriptional data of human primary DC subpopulations suggest that the type 1 CLR CLEC10A [CD301, macrophage galactose-type C-type lectin (MGL), and CLECSF14] might be an interesting candidate expressed on human CD1c^+^ DCs (15, 17, 36) and human CD103^+^SIRPα^+^ DCs, the equivalent of CD1c^+^ DCs in the human gut (16). Although transcriptomic analyses of human primary monocytes revealed human CLEC10A mRNA expression in intermediate monocytes (CD14^++^CD16^+^), only very low protein expression could be detected in these cells (37). Originally, human CLEC10A was identified as a CLR expressed on immature monocyte-derived DCs (moDCs), but not or to a lower extend on mature moDCs (38). It was further demonstrated that the carbohydrate recognition domain of CLEC10A recognizes galactose/*N*-acetylgalactosamine (Tn antigen) (38). In mice, two homologs of CLEC10A can be distinguished (CD301a/MGL1 and CD301b/MGL2), which show different expression patterns and binding specificities (39, 40). MGL1 was specifically present on macrophages, whereas MGL2 is expressed on DCs (41). Taking together, these data imply CLEC10A as a useful marker to distinguish DC subsets (16).

The unique transcriptional expression pattern of CLEC10A in human lymphohematopoietic tissues prompted us to dissect CLEC10A protein expression in blood, thymus, and spleen. We here show a CD1c^+^ DC specific, but organ-irrespective expression profile of CLEC10A. Our data further indicate rapid receptor internalization upon binding of an anti-CLEC10A antibody or a natural ligand (with Tn glycosylated MUC-1 peptide) of the receptor. Furthermore, this ligand in combination with the TLR7/8 ligand R848 led to an enhanced secretion of the cytokines IL-8, IL-10, and TNFα by CD1c^+^ DCs. Due to its unique expression profile and its endocytic activity, we are here proposing that CLEC10A might be not only a very good marker for the distinction of human CD1c^+^ DCs from monocytic lineages in lymphoid tissues but also a suitable receptor for the *in vivo* delivery of antigens to human CD1c^+^ DCs.

## Materials and Methods

### Human Tissue Preparation

Leukocyte reduction cones were retrieved from anonymous healthy adult donors. Thymus samples were retrieved from cardiac surgeries of otherwise healthy children. The sources of spleen samples were patients requiring therapeutic splenectomy. All samples were received under local ethical committee approvals (Ethikkommission der Friedrich-Alexander-Universität Erlangen-Nürnberg), and informed written consents were obtained in accordance with the Declaration of Helsinki.

All tissues were freshly processed as described earlier (15). In brief, thymic and splenic tissues were chopped into small pieces using forceps and scalpel. Then, the tissue was transferred into C-tubes (Miltenyi Biotec), filled with 5 ml RPMI1640, further mechanically disrupted using a Gentle MACS tissue dissociator (Miltenyi Biotec), and enzymatically digested with 400 U/ml collagenase D (Serva) and 100 µg (spleen) or 300 µg (thymus) deoxyribonuclease I (Sigma). After filtering the cell suspension twice, cell suspension of splenic and thymic tissue as well as the leukocyte enriched fraction of human blood was diluted with RPMI1640 and a density gradient centrifugation using Human Pancoll (ρ = 1.077 g/ml; Pan Biotech) was performed as described earlier. After the centrifugation, the interphase containing the mononuclear cells was collected, washed twice with RPMI1640, and used for experiments.

### Microarray Analysis

Published microarray data were analyzed for relative expression of CLEC10A (15). Microarray data are available in the Gene Expression Omnibus database (www.ncbi.nlm.nih.gov/gds) under the accession number GSE77671. Transcriptome data of whole Human Genome Oligo microarray (Agilent) of human CD1c^+^ DCs, CD141^+^ DCs, and pDCs from three blood, spleen, and thymus donors as well as blood monocytes, B cells, and CD4^+^ and CD8^+^ T cells were used. Raw values generated by automated feature extraction have been RMA background corrected and quantile normalized using R (Windows, x64, 3.3.1) (42). Relative expression values were plotted taking advantage of the gplots package of R (43).

### Flow Cytometry

Flow cytometric analyses of single cell suspensions of blood, spleen, and thymus were performed on a BD LSRFortessa and analyzed using FlowJo software (Treestar). 5 × 10^6^ (blood) to 8 × 10^6^ (thymus, spleen) cells were stained with the following antibody cocktail to discriminate different immune cell populations for 15 min at 4°C: CD1c-APC/Cy7 (L161; BioLegend), CD3-BUV395 (UCHT1; BD Biosciences), CD4-BV510 (OKT4; BioLegend), CD8-APC (RPA-T8; eBioscience) or CD8-BUV737 (SK1; BD Biosciences), CD11b-PE/Cy5 (M1/70; BioLegend), CD11c-PE/Cy7 (3.9; BioLegend), CD14-A700 (HCD14; BioLegend), CD19-BV605 (SJ25C1; BioLegend), CD20-BV605 (2H7; BioLegend), CD56-BV421 (5.1H11; BioLegend), CD123-BV650 (6H6; BioLegend), CD141-BV711 (1A4; BD Bioscience), CD303-PerCP/Cy5.5 (201A; BioLegend), CLEC10A-PE or -APC (H037G3; BioLegend), HLA-DR-PE-CF594 (G46-6; BD Bioscience), and NKp46–BV421 (9E2; BioLegend). As appropriate isotype control for CLEC10A-PE and -APC, the cells were stained with a PE- or APC-labeled mouse IgG2a isotype control (MOPC-173; BioLegend). For some experiments, CLEC10A was stained using 10 µg/ml of an FITC-coupled MUC-1 peptide (βAla-GVTSAPDTRPAPGSTAPPAHGVT-NH_2_) that was glycosylated with N-acetylgalactosamine (Tn antigen) at Serine 4 and Threonine 15 [referred to as MUC-1-(Tn)_2_] or a non-glycosylated FITC- βAla-MUC-1 peptide as control (referred to as MUC-1). Synthesis and characterization of the compounds have been described earlier (44). After staining for 30 min on ice, the cells were washed and stained for different immune cell populations as before. After washing the cells, they were resuspended in PBS + 2% human sera + 0.1 mg/ml 4′,6-diamidino-2-phenylindole and acquired using a BD LSRFortessa.

### Internalization Assay

For the internalization assay, 5 × 10^6^ cells were stained with anti-CLEC10A-PE (H037G3; BioLegend) or an appropriate isotype control (mouse IgG2a, MOPC-173; BioLegend) for 15 min on ice. Then, the cells were incubated for different time points at 37°C (5, 15, 30, and 60 min) or kept on ice for 60 min. In a next step, surface CLEC10A was stained with an anti-PE antibody (polyclonal goat IgG; Novus Biologicals) and after washing with an anti-goat-A647 (polyclonal donkey IgG; Southern Biotech). In a last step, the cells were stained for the discrimination of different immune cell populations as described under flow cytometry and samples acquired using a BD LSRFortessa.

### Generation of moDCs

PBMCs were isolated by gradient-density centrifugation. 5 × 10^7^ PBMCs in 10 ml DMEM were adhered to a human IgG-coated tissue culture dish (BD Falcon) and incubated for 1 h at 37°C. Then, the medium was replenished with 10 ml moDC medium [RPMI-1640/containing 1% penicillin/streptomycin, 1% l-glutamine, 1% HEPES, and 2% human sera type AB (Lonza)]. On the next day, the medium was replenished with fresh moDC medium containing 800 U/ml GM-CSF and 50 U/ml IL-4. On day 3 and day 5, 4 ml of fresh moDC medium was added containing 800 U/ml GM-CSF/50 U/ml IL-4 and 400 U/ml GM-CSF/25 U/ml IL-4, respectively. On day 6, half of the cells were matured using a maturation cocktail consisting of 13.2 ng/ml IL-1β, 10,000 U/ml IL-6, 10 ng/ml TNFα, and 1 µg/ml prostaglandin E_2_ for 24 h. IL-1β, IL-6, and TNFα were purchased from PeproTech and prostaglandin E_2_ from Sigma Aldrich.

### Enrichment of DCs

In order to perform cell sorts and for internalization experiments using thymic DCs, DCs were enriched prior to the experiments. Therefore, blood DCs were enriched with the EasySep Pan-DC Pre-Enrichment Kit (Stemcell Technologies) as described in the manual of the manufacturer. Briefly, PBMCs were diluted in PBS + 2% human sera + 1 mM EDTA and stained with anti-human CD32 (Fc gamma RII) Blocker (Stemcell Technologies) and the EasySep Pan-DC Pre-Enrichment Cocktail for 45 min at room temperature (RT). Then, magnetic Dextran Rapidspheres were added, and the cells were incubated for 10 min at RT followed by incubation for 5 min inside “The Big Easy” EasySep Magnet (Stemcell Technologies). The unlabeled DCs were poured off into a new tube and stained for cell sorts.

Thymic DCs were enriched using biotinylated antibodies against thymocytes (CD7, clone eBio124-1D1; eBioscience), T cells (CD3, SK7, BioLegend; CD8, RPA-T8, BioLegend), B cells (CD19, HIB19; BD Bioscience), and NK cells (CD56, MEM-188; BioLegend), and MojoSort Streptavidin Nanobeads (BioLegend). The cell suspension was stained with the cocktail of biotinylated antibodies in PBS + 2% human sera + 1 mM EDTA for 30 min on ice. Then, the cells were washed, resuspended in PBS + 2% human sera + 1 mM EDTA, and MojoSort Streptavidin Nanobeads added. After incubation for 15 min on ice, the tube was transferred into “The Big Easy” EasySep Magnet and incubated for 5 min at RT. Next, the unlabeled DCs were poured off into a new tube and used for further experiments.

### Cell Sorting of Human DC Subpopulations

Dendritic cell-enriched cell suspensions were stained with an antibody cocktail consisting of the following antibodies in PBS + 2% human sera for 30 min on ice: CD1c-APC/Cy7 (L161; BioLegend), CD3-BUV395 (UCHT1; BD Bioscience), CD11b-A700 (M1/70; BioLegend), CD11c-PE/Cy7 (3.9; BioLegend), CD14-A700 (HCD14; BioLegend), CD19-V450 (HIB19; BD Bioscience), CD20-eFluor 450 (2H7; eBioscience), CD56-BV421 (5.1H11; BioLegend), CD123–BV605 (6H6; BioLegend), CD141-BV711 (1A4; BD Bioscience), CD303-PerCP/Cy5.5 (201A; BioLegend), HLA-DR-PE-CF594 (G46-6; BD Bioscience), and NKp46-BV421 (9E2; BioLegend). After washing the cells, they were resuspended in PBS + 2% human sera + 0.1 mg/ml 4′,6-diamidino-2-phenylindole and cell sorted using a BD FACSAria II cell sorter into CD1c^+^ DCs (CD3^−^CD11b^−^CD14^−^CD19^−^CD20^−^CD56^−^CD123^−^NKp46^−^HLA–DR^+^CD11c^+^CD1c^+^), CD141^+^ DCs (CD1c^−^CD3^−^CD11b^−^CD14^−^CD19^−^CD20^−^CD56^−^CD123^−^NKp46^−^HLA-DR^+^CD11c^+^CD141^+^), pDCs (CD1c^−^CD3^−^CD11b^−^CD14^−^CD19^−^CD20^−^CD56^−^NKp46^−^HLA-DR^+^CD123^+^CD303^+^), and monocytes (CD3^−^CD19^−^CD20^−^CD56^−^CD123^−^NKp46^−^HLA-DR^+^CD11b^+^CD14^+^). The purity of sorted cell populations was reanalyzed and routinely above 95%.

### Stimulation of Sorted DCs

Cell-sorted DC subpopulations and monocytes were resuspended in DC medium (RPMI1640 + 5% human sera + 5% Pannexin NTA + 5% Pannexin NTS + 1% glutamine + 1% sodium pyruvate + 1% penicillin/streptomycin) to a concentration of 2 × 10^5^ cells/ml. Cells were cultured in sterile 96-well plates (v-bottom) and stimulated either with one of the TLR ligands pIC (5 µg/ml) or R848 (5 µg/ml), the CLEC10A ligand MUC-1-(Tn)_2_ (10 µg/ml) or a non-glycosylated MUC-1 control peptide (10 µg/ml), or a combination of both. TLR ligands were purchased from Invivogen and reconstituted in water. After 12 h of stimulation, the cells were analyzed by flow cytometry for the expression of CLEC10A (CLEC10A-APC, clone: H037G3, BioLegend) and activation markers (CD40-PE, clone: 5C3, BioLegend; CD86-PE-CF594, 2331(FUN-1), BD Biosciences) together with sorted DCs and monocytes of the very same donor, which were kept on ice until analysis. Supernatants of stimulated cells were harvested and stored at −80°C until analysis with the LEGENDplex Human Anti-Virus Response Panel (BioLegend). Acquisition of the samples was performed using a BD LSRFortessa and data analyzed using FlowJo (CLEC10A, CD40, CD86) or LEGENDplex software (VigeneTech; cytokine secretion).

### Intracellular Signaling in Sorted CD1c^+^ DCs

CD1c^+^ DCs were sorted from PBMCs of healthy donors, resuspended in DC medium to a final concentration of 2 × 10^5^ cells/ml and stimulated either with 10 µg/ml MUC-1-(Tn)_2_ or non-glycosylated MUC-1 in combination with 5 µg/ml R848 and incubated at 37°C for different time points (0, 5, 15, 30, and 60 min). Afterward, the cells were washed and fixed with Cytofix/Cytoperm Buffer (BD Bioscience) for 20 min on ice. Then, the cells were washed and permeabilized with Perm Buffer III (BD Bioscience) for 30 min on ice. After washing, the cells were stained with BV421 Mouse Anti-Human NF-κB p65 (pS529), Alexa Fluor^®^ 647 Mouse anti-JNK (pT183/pY185), Alexa Fluor^®^ 488 Mouse Anti-CREB (pS133)/ATF-1 (pS63), PE-CF594 Mouse Anti-p38 MAPK (pT180/pY182), PE-C7 Mouse anti-ERK1/2 (pT202/pY204), and PE Mouse anti-Human IKKγ (pS376) for 30 min at RT. All antibodies were purchased from BD Bioscience. The cells were washed and then acquired using a BD LSRFortessa.

## Results

### CLEC10A Is Specifically Expressed on Human CD1c^+^ DCs in Various Lymphoid Tissues

**Figure 1:**
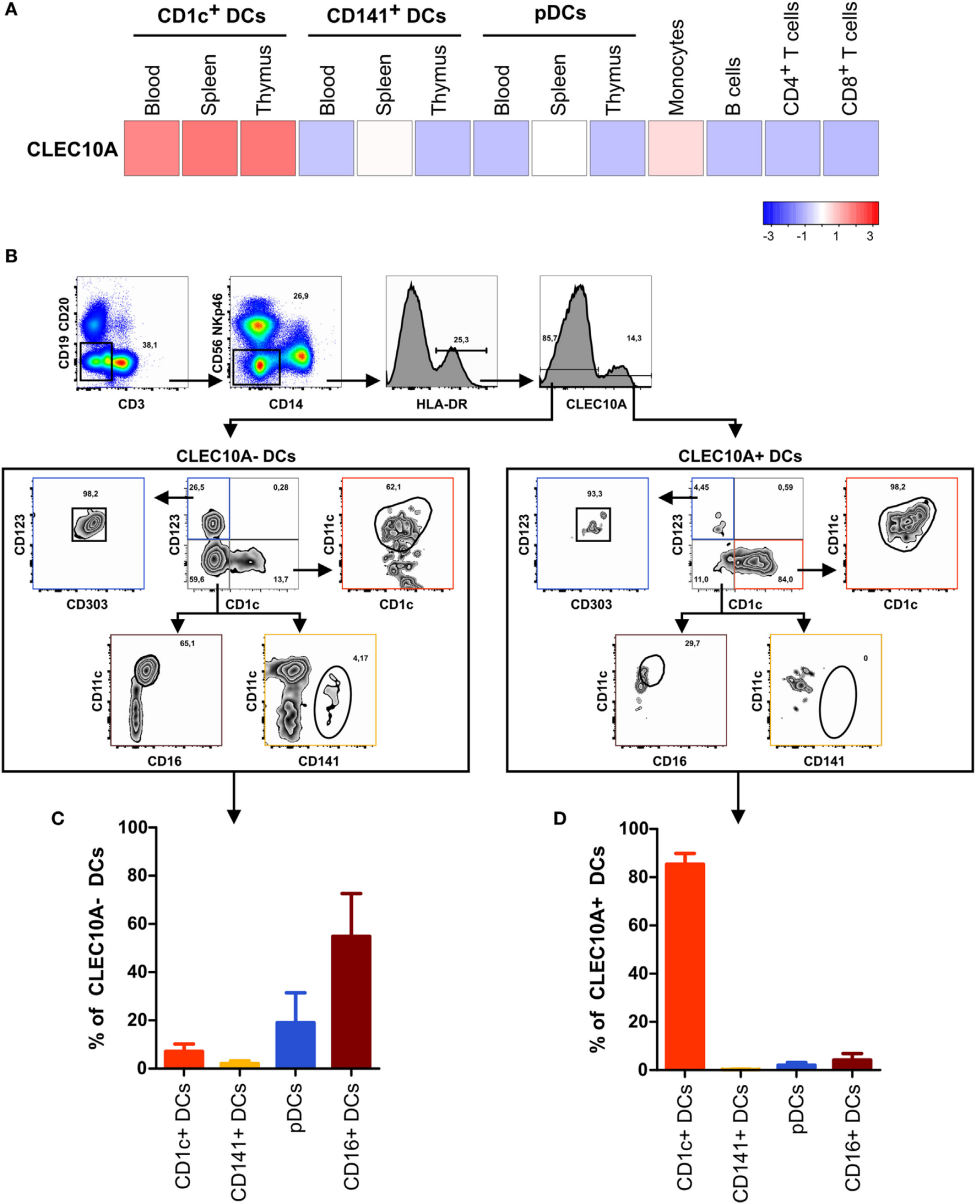
The CLEC10A^+^ dendritic cell (DC) compartment is predominantly composed of CD1c^+^ DCs in human blood. **(A)** The expression of CLEC10A was analyzed by microarray analysis. Previously published microarray data (15) were used (GEO accession number GSE77671). For these microarray data, DC subpopulations from each three blood, spleen, or thymus donors as well as blood monocytes, B cells, CD4^+^ and CD8^+^ T cells were sorted by flow cytometry, and whole human genome microarray (Agilent) analyses were performed (15). The heat map shows the relative expression of CLEC10A transcripts. **(B–D)** PBMCs of a healthy donor were stained with a PE-labeled anti-CLEC10A antibody as well as antibodies against different immune cell populations and analyzed by flow cytometry. **(B)** After exclusion of doublets and dead cells (not shown), lineage^+^ (CD3, CD14, CD19, CD20, CD56, and NKp46) cells were excluded. In the remaining cells, HLA-DR^+^ cells were selected and CLEC10A^+^ and CLEC10A^−^ cells gated. The CLEC10A^+^ and CLEC10A^−^ cells were then analyzed for the proportion of CD1c^+^ DCs (CD1c^+^CD11c^+^CD123^−^), CD141^+^ DCs (CD141^+^CD11c^±^CD1c^−^CD123^−^), plasmacytoid DCs (pDCs) (CD123^+^CD303^+^CD1c^−^), and CD16^+^ DCs (CD11c^+^CD16^+^CD1c^−^CD123^−^). One representative donor is shown. **(C,D)** The percentages of CD1c^+^ DCs, CD141^+^ DCs, pDCs, and CD16^+^ DCs of **(C)** CLEC10A^−^ and **(D)** CLEC10A^+^ cells were calculated and plotted as bar graphs (mean ± SD; *n* = 3).

**Figure 2:**
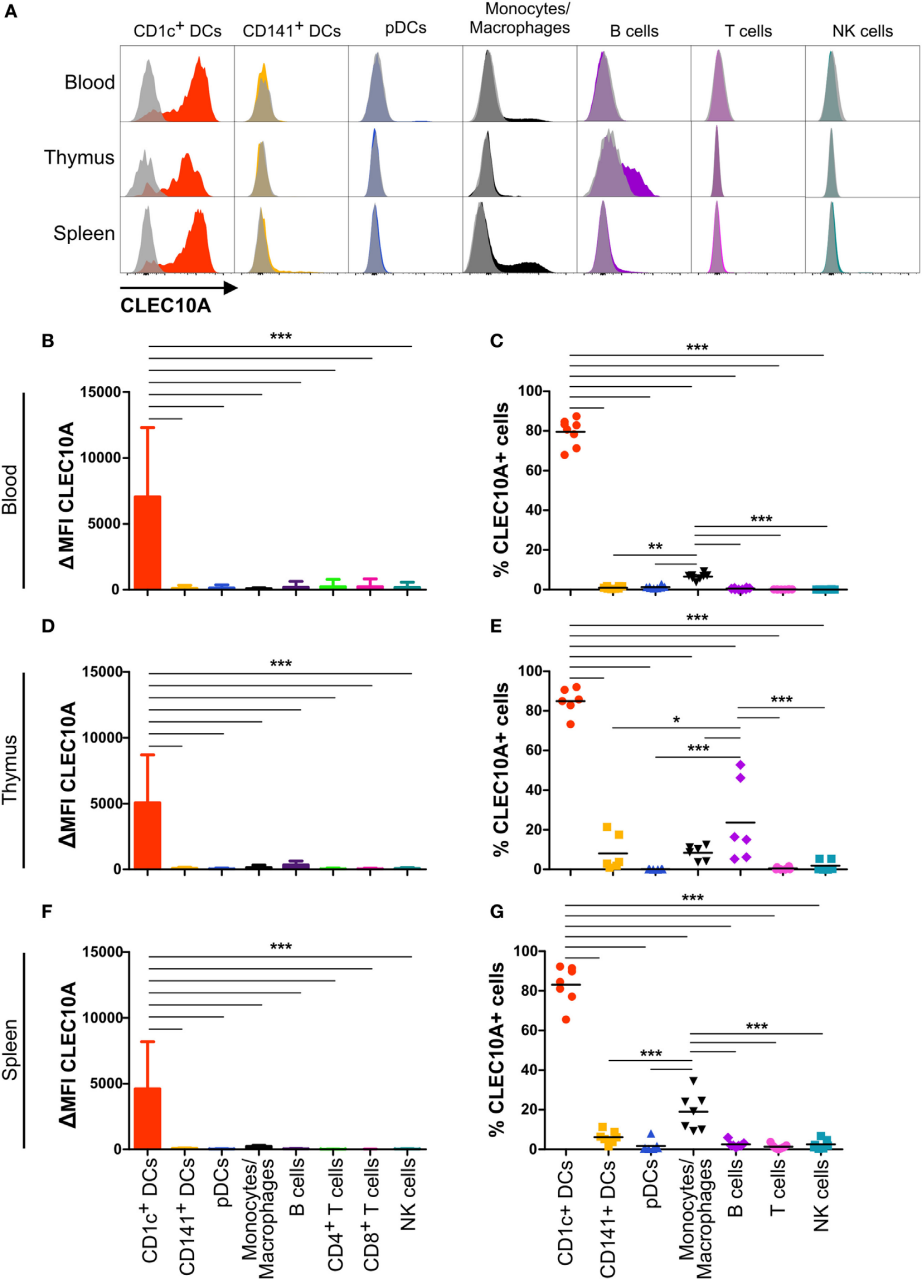
CLEC10A is mainly expressed on human CD1c^+^ dendritic cells (DCs) in different tissues in the steady state. The expression of CLEC10A was analyzed by flow cytometric analysis on human DC subpopulations in blood, thymus, and spleen. **(A)** Depicted are overlay histograms of single cell suspensions of one representative donor each of blood, thymus, and spleen analyzed by flow cytometry for an anti-CLEC10A antibody (colored) and appropriate isotype control (gray, transparent). Bar graphs in **(B,D,F)** show the ΔMFI for CLEC10A (median ± SD, blood *n* = 8; thymus *n* = 6; spleen *n* = 7). **(C,E,G)** In each leukocyte population, the CLEC10A^+^ cells were determined using an appropriate isotype control and plotted as a scatter plot with mean as horizontal line. Significant differences in the **(B,D,F)** expression of CLEC10A or **(C,E,G)** percentages of CLEC10A^+^ cells were calculated by one-way ANOVA with Bonferroni posttest using GraphPad Prism (*p < 0.05, **p < 0.01, and ***p < 0.001).

In our recently performed transcriptional analysis of human lymphohematopoietic organ DC subpopulations (blood, thymus, and spleen), we demonstrated a strong expression of the CLEC10A mRNA in CD1c^+^ DCs in all tested tissues (Figure 1A) (15). In line with an earlier report about intermediate monocytes (CD14^++^CD16^+^), we could confirm a low CLEC10A mRNA expression in monocytes (Figure 1A) (37). To prove that CLEC10A is also expressed on protein level, we performed flow cytometric analyses on single cell suspensions of human blood. We found that the CLEC10A^+^ lineage (CD3, CD14, CD19, CD20, CD56, and NKp46) negative cells consisted almost exclusively of CD1c^+^ DCs (Figures 1B,D). In contrast, the CLEC10A^−^ lineage negative cells consisted mainly of CD16^+^ DCs, CD141^+^ DCs, and pDCs (Figures 1B,C). In addition, a low amount of CD1c^+^ DCs were represented in the CLEC10A^−^ DC compartment (Figure 1C). We further extended our analyses to thymus and splenic tissue and analyzed the expression of CLEC10A on other cells of the immune system (Figure 2). Remarkably, also in these lymphoid tissues CLEC10A demonstrated a selective expression on human CD1c^+^ DCs comparable to the blood (Figures 2A,B,D,F). Only to a low extent we found CLEC10A protein expression on thymic B cells and blood as well as splenic monocytes/macrophages (Figure 2). Notably, no expression could be detected on CD141^+^ DCs, pDCs, T cells, or NK cells (Figure 2). Taken together, we found that CLEC10A was expressed on about 80% of CD1c^+^ DCs in all tested tissues, while it was only expressed on about 5% of blood monocytes, 20% of thymic B cells, and 20% of splenic monocytes/macrophages (Figures 2C,E,G). In summary, our data suggest a prevalent expression profile of CLEC10A protein on human CD1c^+^ DCs.

### CLEC10A Is Rapidly Internalized Into Human CD1c^+^ DCs

**Figure 3:**
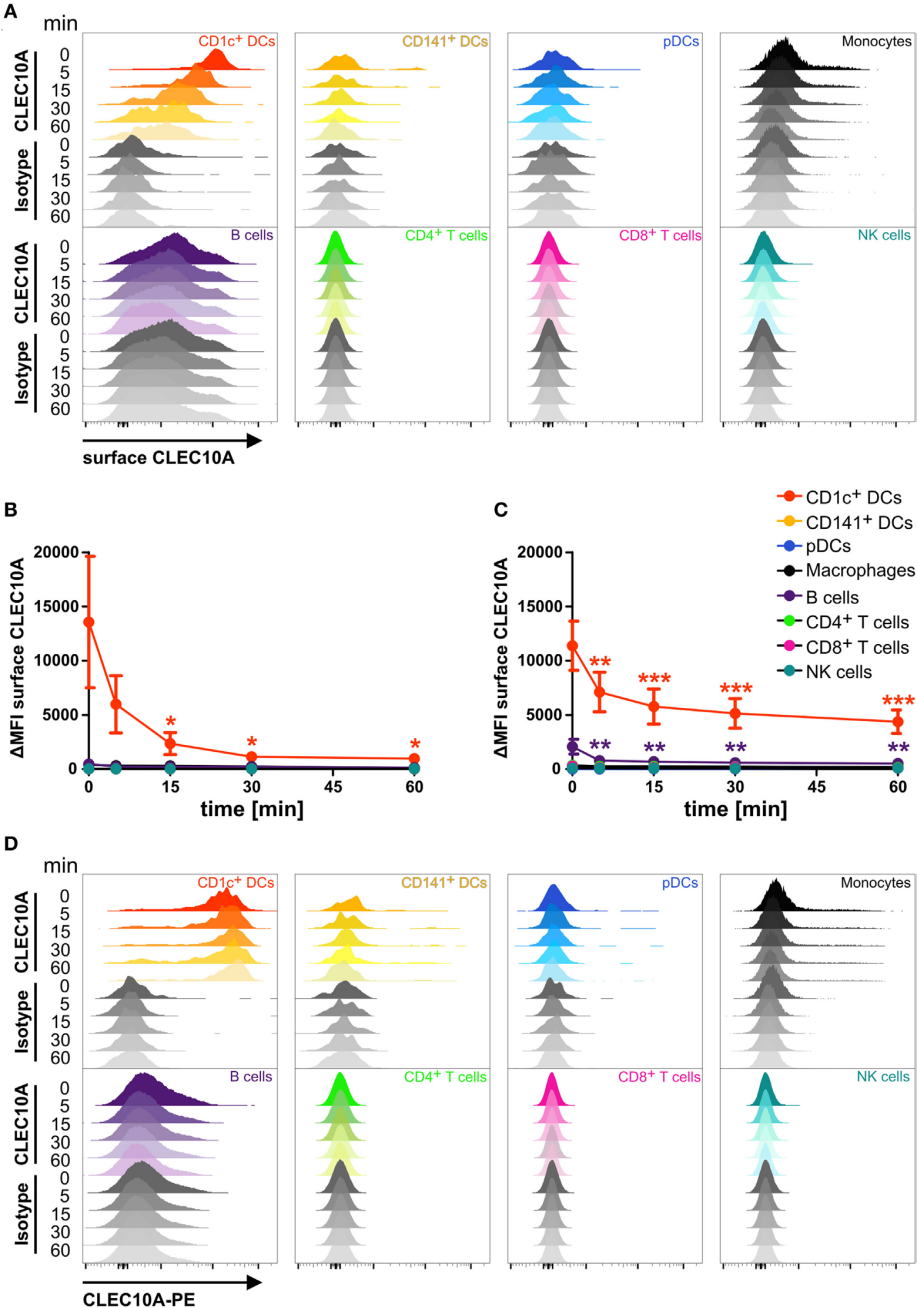
CLEC10A is rapidly internalized into blood and thymic CD1c^+^ dendritic cells (DCs). Single cell suspensions of blood **(A,B,D)** and thymus **(C)** were incubated with a PE-coupled anti-CLEC10A antibody or an appropriate isotype control at 4°C for 15 min, washed, and resuspended in PBS containing 2% human sera. Subsequently, the cells were incubated at 37°C for different time points (0, 5, 15, 30, and 60 min) to allow for the internalization of the primary PE-coupled antibody. Afterward, the cells were stained for surface CLEC10A by an anti-PE secondary antibody (polyclonal goat IgG) and an A647-labeled tertiary anti-goat antibody. Then, the cells were stained for different immune cell populations. 2.5 × 10^6^ cells were acquired using a BD LSRFortessa and analyzed using FlowJo. **(A)** Overlay histograms show surface expression of CLEC10A for CD1c^+^ DCs (red), CD141^+^ DCs (yellow-orange), plasmacytoid DCs (pDCs) (blue), monocytes (black), B cells (purple), CD4^+^ T cells (green), CD8^+^ T cells (pink), and NK cells (cyan). **(B,C)** PBMCs **(B)** and thymic **(C)** cells were analyzed as in **(A)**. Plot shows mean ± SD of surface CLEC10A (MFI_CLEC10A_ − MFI_isotype control_) of three **(C)** or five **(B)** donors. Statistical analysis was performed using one-way ANOVA with Dunnett posttest for each cell population (ΔMFI 0 min as control; *p < 0.05, **p < 0.01, and ***p < 0.001). **(D)** Overlay histograms show the PE-signal of the primary PE-coupled anti-CLEC10A antibody or the appropriate isotype control for the same donor as in **(A)**.

**Figure 4:**
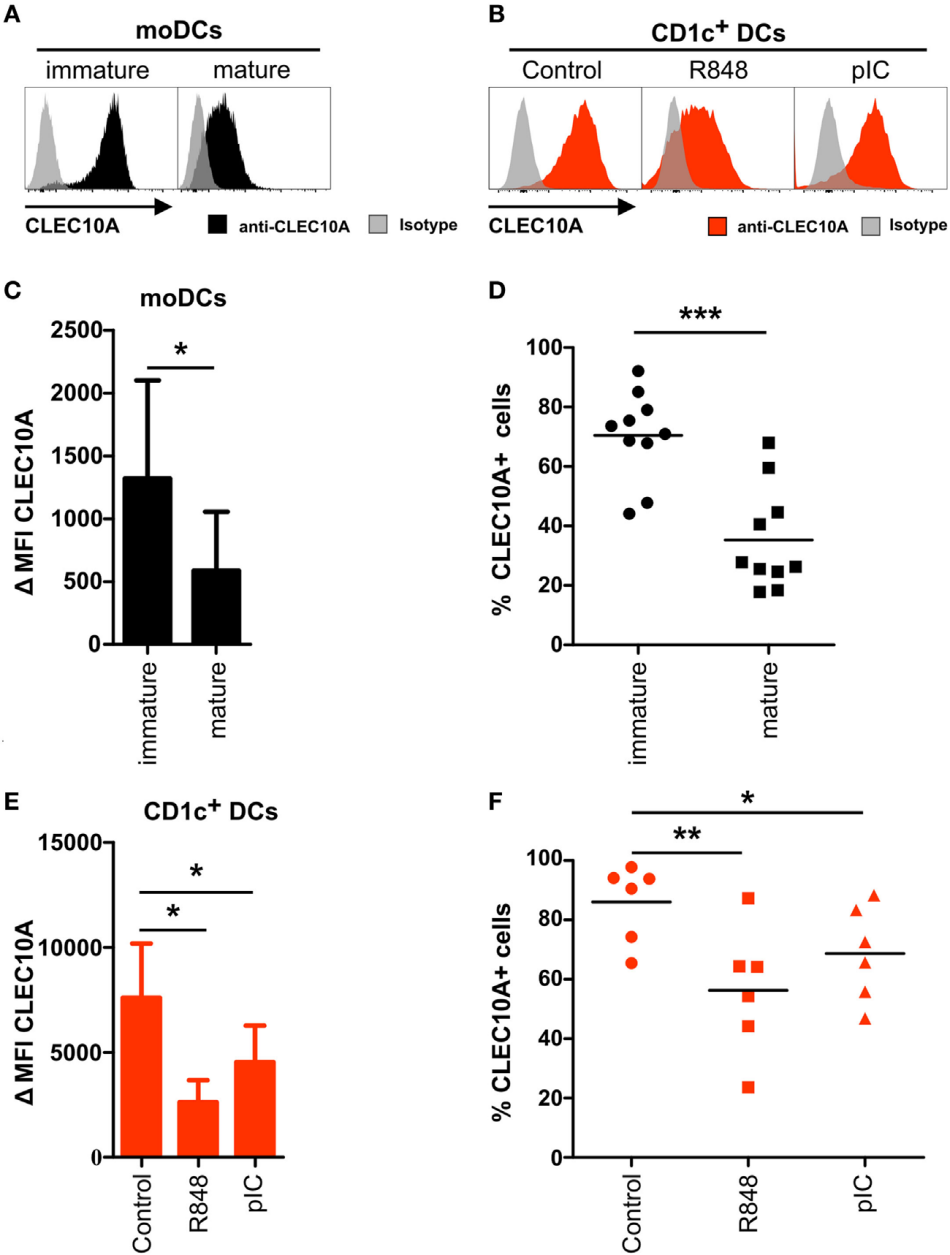
CLEC10A is downregulated on activated human CD1c^+^ dendritic cells (DCs). **(A,C,E)** Monocyte-derived DCs were generated from blood monocytes of a healthy donor. After 6 days, half of the cells were matured using a maturation cocktail consisting of IL-1β, IL-6, TNFα, and PGE_2_ for 24 h. **(B,D,F)** Blood CD1c^+^ DCs (red) were sorted from PBMCs of a healthy donor and subsequently stimulated either with R848 (5 µg/ml), pIC (5 µg/ml), or cultured in DC medium for 12 h at 37°C. Afterward, the cells were stained with an APC-labeled anti-CLEC10A antibody or an appropriate isotype control and analyzed using a BD LSRFortessa and FlowJo software. **(A,B)** Overlay histograms demonstrate a representative donor for **(A)** moDCs (black: CLEC10A; gray-transparent: isotype control) and **(B)** CD1c^+^ DCs (red: CLEC10A; gray-transparent: isotype control). **(C,E)** Bar graphs show the expression of CLEC10A [mean ± SD of ΔMFI (MFI_CLEC10A_ − MFI_Isotype control_)] on **(C)** moDCs (*n* = 10) and **(E)** CD1c^+^ DCs (*n* = 6). **(D,F)** CLEC10A^+^ **(D)** moDCs (black symbols) and **(F)** CD1c^+^ DCs (red symbols) were determined using an appropriate isotype control and blotted as scatter plots. Significant differences in the **(C,E)** expression of CLEC10A or **(D,F)** percentages of CLEC10A^+^ cells were calculated using *t* test (*p < 0.05, **p < 0.01, and ***p < 0.001).

As a potential receptor for antigen-targeting approaches, it is necessary that antibodies bound to its specific receptor can internalize into the cell. To investigate the endocytic activity of the CLEC10A receptor, we performed an internalization assay. We therefore stained single cell suspensions of blood and thymic tissue with a PE-coupled anti-CLEC10A antibody. After binding of the antibody, the cells were washed and incubated at 37°C for different time points to allow the internalization of the antibody bound to CLEC10A. To distinguish between antibodies that were internalized from remaining antibodies at the surface of the cells, we stained the cells with a secondary antibody recognizing the PE fluorochrome coupled to the anti-CLEC10A antibody. For controlling the internalization efficacy, the secondary surface-bound antibody was visualized by a tertiary A647-labeled antibody directed against the Fc part of the secondary antibody. In cells that were kept on ice, the PE-labeled primary anti-CLEC10A antibody remained on the surface and thereby the cells displayed a strong staining for the A647-labeled tertiary antibody (Figure 3). With increasing incubation time, the A647 signal was reduced (Figures 3A–C), whereas the PE signal of the primary PE-labeled anti-CLEC10A antibody was stable over the whole incubation time (Figure 3D). Our data indicate that already after 5 min at 37°C large amounts of the PE-labeled anti-CLEC10A antibody were internalized into CD1c^+^ DCs. We did not observe other cells, which expressed or internalized the antibody except for a low internalization into thymic B cells (Figure 3). Interestingly, whereas blood CD1c^+^ DCs completely internalized the anti-CLEC10A antibody within 30 min (Figure 4B), about 50% of the signal remained on the surface of thymic CD1c^+^ DCs (Figure 3C). Thus, our data suggest a strong endocytic activity of CLEC10A upon antibody binding.

### CLEC10A Is Downregulated on Activated CD1c^+^ DCs

Human CLEC10A was identified as a CLR expressed on immature moDCs. To place our data into the context of previous findings, we performed flow cytometric analyses on immature and mature moDCs. Accordingly, we could detect a low to intermediate surface expression of CLEC10A on immature moDCs (Figures 4A,C). In line with earlier published data, displaying an absent expression of CLEC10A-mRNA in mature moDCs (38), we found that after stimulation of moDCs with a maturation cocktail consisting of IL-1β, IL-6, TNFα, and prostaglandin E2, the expression of CLEC10A was significantly reduced, but not absent, on mature moDCs (Figures 4A,C,D).

To investigate potential expression changes of CLEC10A on activated human CD1c^+^ DCs, we stimulated sorted human blood CD1c^+^ DCs with TLR ligands R848 (TLR7/8) or pIC (TLR3) and analyzed the cells by flow cytometry. In line with the results obtained with moDCs and independent on the used stimulus, 12 h upon TLR ligand stimulation we found a reduced surface expression of CLEC10A on blood CD1c^+^ DCs (Figures 4B,E), in which TLR7/8 stimulation was superior to TLR3 stimulation, as the first demonstrated a stronger reduction of the CLEC10A surface expression. Comparable to data generated from moDCs, TLR stimulated CD1c^+^ DCs remained partially positive for CLEC10A (Figure 4F). Our data suggest a typical behavior of CLEC10A compared to other type I CLRs such as DCIR, DC-Sign, or CLEC9A upon cell stimulation.

### CD1c^+^ DCs Specifically Bind Glycosylated Ligands for CLEC10A

**Figure 5:**
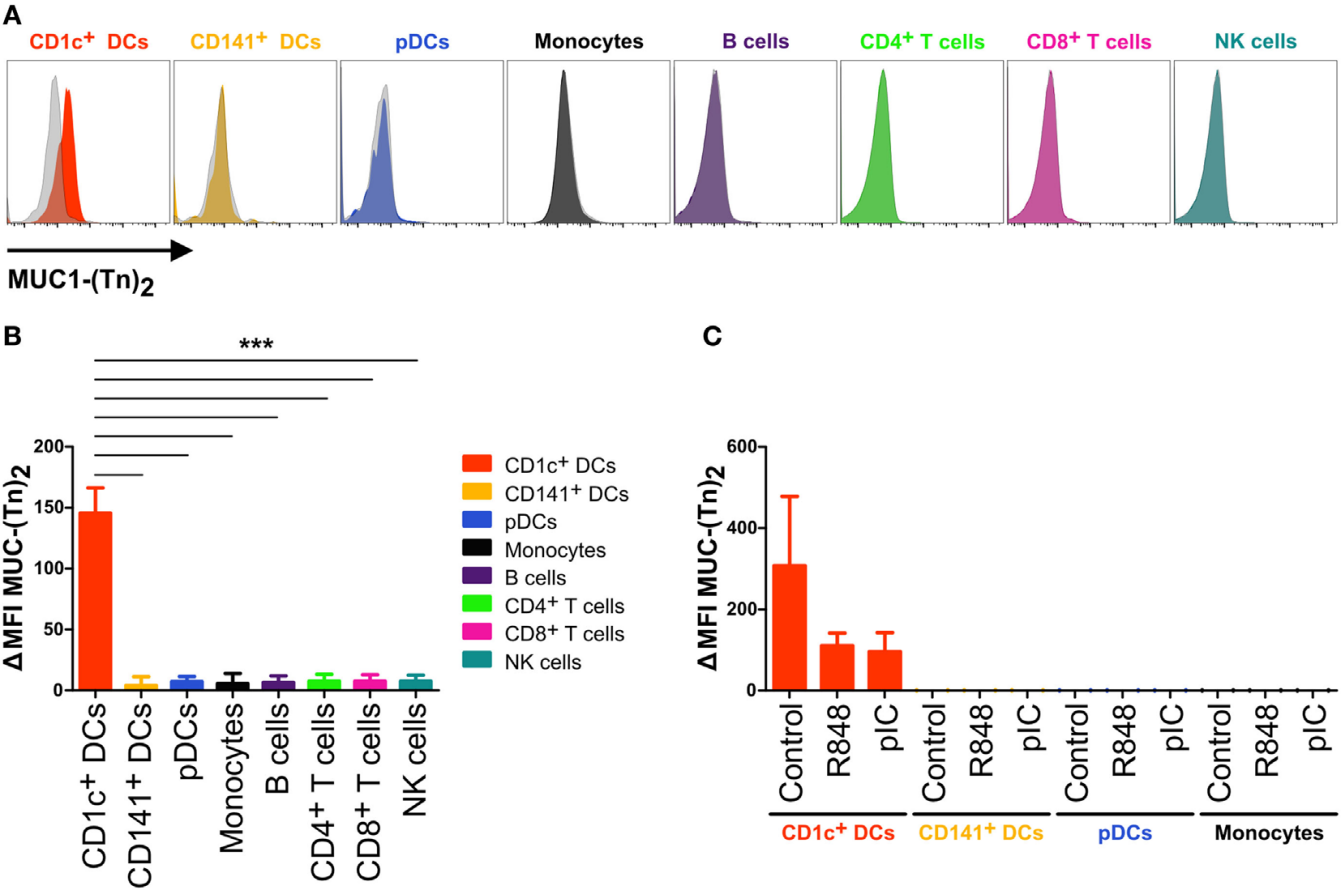
CD1c^+^ dendritic cells (DCs) bind specifically to glycosylated ligands for CLEC10A. **(A,B)** PBMCs of healthy human donors were incubated with 10 µg/ml of a glycosylated or non-glycosylated MUC-1 peptide. After washing with FACS buffer twice, the cells were stained for different immune cell populations. 1.5 × 10^6^ cells were acquired using a BD LSRFortessa and analyzed using FlowJo software. **(A)** Overlay histograms demonstrate the binding of glycosylated MUC-1-(Tn)_2_ (colored) and non-glycosylated MUC-1 (gray-transparent) to CD1c^+^ DCs (red), CD141^+^ DCs (yellow-orange), plasmacytoid DCs (pDCs) (blue), monocytes (black), B cells (purple), CD4^+^ T cells (light-green), CD8^+^ T cells (pink), and NK cells (cyan). **(B)** Bar graphs show the ΔMFI (ΔMFI = MFI MUC-1-(Tn)_2_ − MFI MUC-1) for the binding of Tn antigen to CLEC10A for the same cell populations as in **(A)**. Significant differences in the binding of MUC-1-(Tn)_2_ to cell populations were calculated using one-way ANOVA with Bonferroni posttest (*p < 0.05, **p < 0.01, and ***p < 0.001). **(C)** CD1c^+^ DCs (red), CD141^+^ DCs (yellow-orange), pDCs (blue), and monocytes (black) of PBMCs of healthy donors were sorted using a FACS Aria II and subsequently incubated with 10 µg/ml FITC-coupled MUC-1-(Tn)_2_ or MUC-1 and stimulated with either 5 µg/ml R848, 5 µg/ml pIC, or left untreated. After 12 h at 37°C, the cells were analyzed for the binding of MUC-1-(Tn)_2_ by flow cytometry. Bar graphs show the ΔMFI of MUC-1-(Tn)_2_.

In order to better define the CLEC10A-specific expression profile, we performed FACS staining experiments applying a previously described bivalent ligand of CLEC10A (44). The use of such natural ligands is not only of importance for the identification of interaction partners and cell activation, natural ligands can also be used for the delivery of antigens to endocytic receptors as it was demonstrated for the chemokine ligand receptor pair XCL1and XCR1 (45, 46). Thus, PBMCs of healthy donors were incubated either with 10 µg/ml of an FITC-coupled MUC-1 peptide (sequence: βAla-GVTSAPDTRPAPGSTAPPAHGVT-NH_2_) that was glycosylated with N-acetylgalactosamine (Tn antigen) at Serine 4 and Threonine 15 (referred to as CLEC10A ligand MUC-1-(Tn)_2_) or with 10 µg/ml of a non-glycosylated FITC-coupled MUC-1 peptide (referred to as MUC-1) (44). After ligand staining, the cells were incubated with antibodies for the detection of different immune cell populations and analyzed by flow cytometry. Analogously to the expression profile we have seen upon application of an anti-CLEC10A antibody, we detected a specific binding of the glycosylated form of MUC-1 [MUC-1-(Tn)_2_] to CD1c^+^ DCs (Figure 5). No other immune cell populations, such as T cells, B cells, NK cells, monocytes, nor CD141^+^ DCs, and pDCs demonstrated an interaction with the glycosylated MUC-1-(Tn)_2_ (Figures 5A,B).

As we found that upon TLR ligand activation of CD1c^+^ DCs the expression of CLEC10A was downregulated, we next investigated CLEC10A ligand binding on activated CD1c^+^ DCs. Therefore, we sorted CD1c^+^ DCs together with CD141^+^ DCs, pDCs, and monocytes from PBMCs of healthy donors and stimulated them simultaneously with the glycosylated or the non-glycosylated MUC-1 peptide [MUC-1-(Tn)_2_ or MUC-1] in combination with one of the TLR ligands R848 or pIC. We found that CD1c^+^ DCs incubated without a TLR ligand bound high amounts of MUC-1-(Tn)_2_, whereas CD1c^+^ DCs stimulated with either R848 or pIC showed a lower binding of MUC-1-(Tn)_2_ (Figure 5C). This is in accordance with the induced downregulation of CLEC10A upon stimulation with the TLR ligands R848 and pIC (Figure 4).

### Stimulation of CD1c^+^ DCs With CLEC10A Ligands Enhances TLR-Induced IL-8, IL-10, and TNFα Secretion

**Figure 6:**
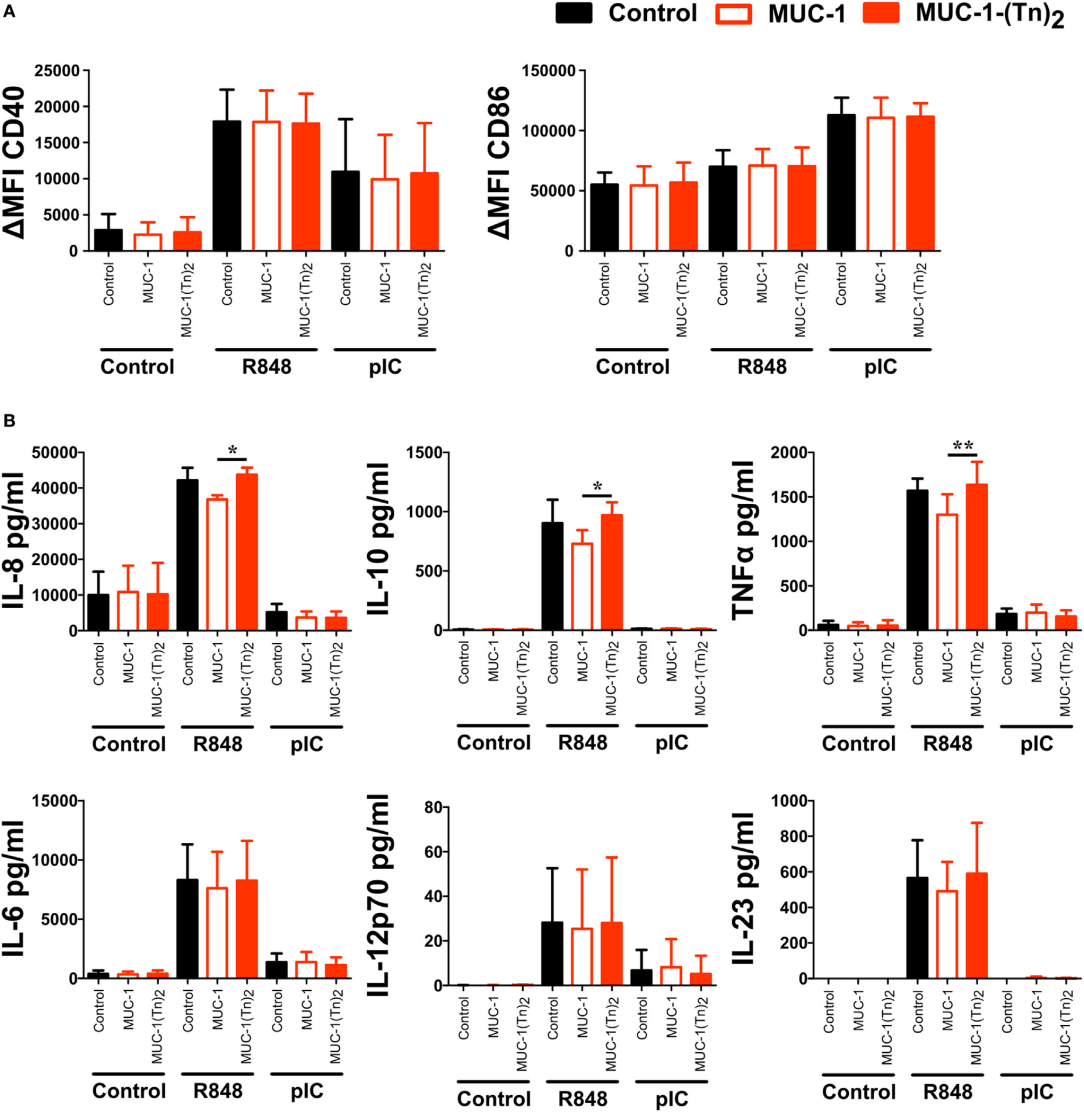
Stimulation of CD1c^+^ dendritic cells (DCs) with MUC-1-(Tn)_2_ enhances TLR-induced secretion of IL-8, IL-10, and TNFα. CD1c^+^ DCs were sorted from PBMCs of healthy donors and stimulated with 10 µg/ml MUC-1-(Tn)_2_ or MUC-1 in the presence of 5 µg/ml R848 or 5 µg/ml pIC or in medium alone. After 12 h of stimulation, **(A)** cells were analyzed for the expression of the co-stimulatory molecules CD40 and CD86 and **(B)** the supernatant analyzed for the secretion of the cytokines IL-8, IL-10, TNFα, IL-6, and IL-12p70 with the LEGENDplex Human Anti-Virus Response Panel (BioLegend). Bar graphs show **(A)** ΔMFI ± SD (*n* = 4) and **(B)** mean ± SD (*n* = 4). *p* values were calculated using GraphPad Prism (paired *t*-test; *p < 0.05 and **p < 0.01).

As some CLRs have been described to induce signaling upon binding of their ligands, we were interested to understand whether stimulation of CD1c^+^ DCs with the CLEC10A ligand MUC-1-(Tn)_2_ would induce signaling and thereby activation and cytokine secretion by the DCs. Therefore, CD1c^+^ DCs were sorted from PBMCs of healthy donors and incubated with glycosylated MUC-1-(Tn)_2_ or the non-glycosylated MUC-1 in the presence or absence of the TLR ligands R848 or pIC. In the absence of further TLR ligand stimulation, we found no activation of CD1c^+^ DCs by the glycosylated MUC-1-(Tn)_2_ or the non-glycosylated MUC-1 (Figure 6A). As shown earlier (47, 48), stimulation with R848 or pIC induced high expression of the co-stimulatory molecules CD40 and CD86, which was not enhanced by binding of MUC-1-(Tn)_2_ to CLEC10A (Figure 6A). We further investigated, if this glycosylated ligand might influence the cytokine secretion of CD1c^+^ DCs, as it was shown that signaling *via* CLEC10A can enhance TLR-induced secretion of cytokines by moDCs (49). We therefore analyzed the supernatants of CD1c^+^ DCs for IL-6, IL-8, IL-10, IL-12p70, IL-23, and TNFα. Neither the incubation with the glycosylated MUC-1-(Tn)_2_ nor the non-glycosylated MUC-1 alone induced strong cytokine secretion by CD1c^+^ DCs (Figure 6B). However, we found a slightly, but statistically significant increased secretion of the cytokines IL-8, IL-10, and TNFα after stimulation with glycosylated MUC-1-(Tn)_2_ in combination with R848, but not pIC (Figure 6B). In contrast, glycosylated MUC-1-(Tn)_2_ did not enhance IL-6, IL-12p70, or IL-23 secretion induced by TLR7/8 or TLR3 stimulation (Figure 6B).

**Figure 7:**
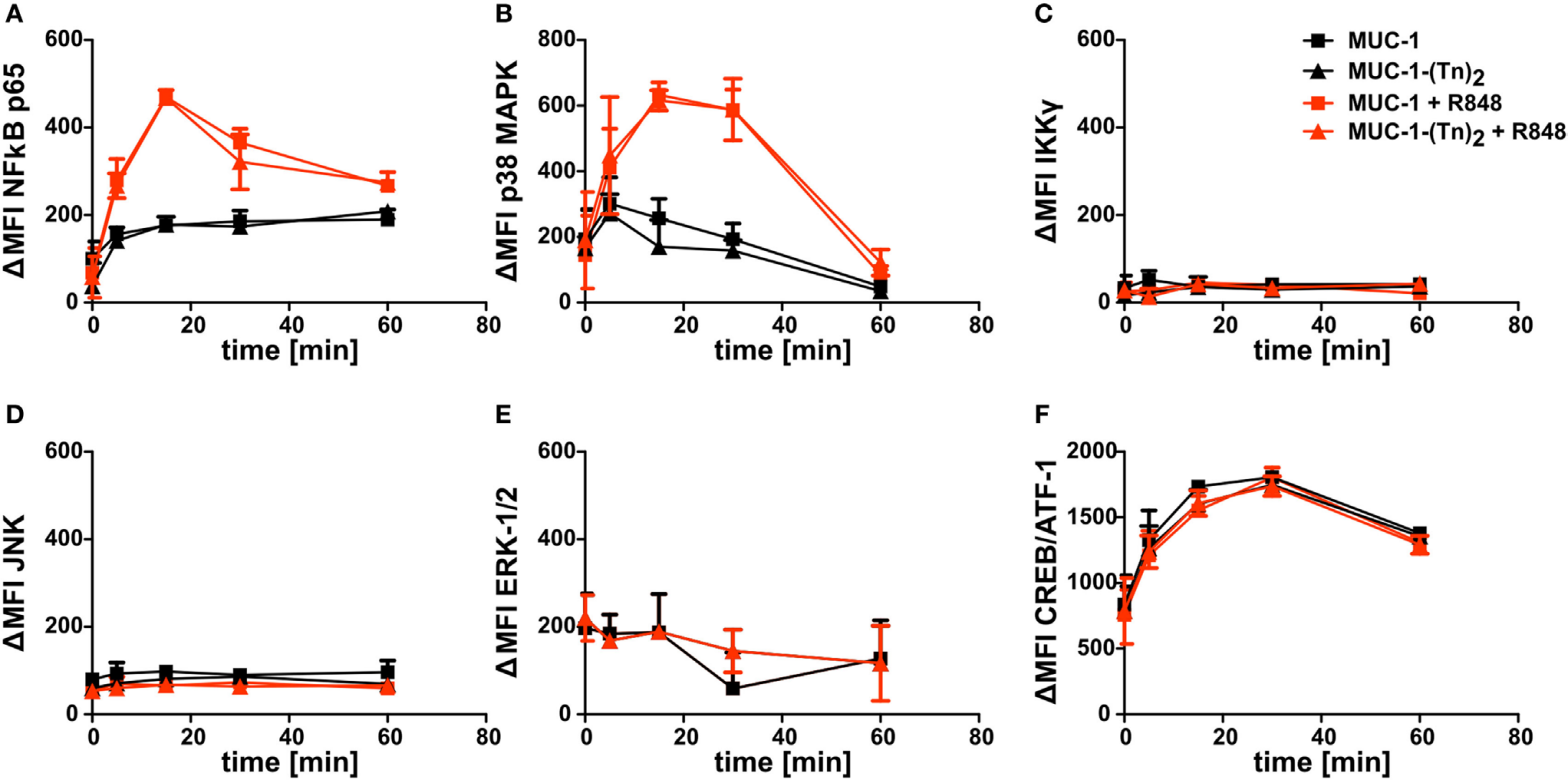
Intracellular signaling in CD1c^+^ dendritic cells (DCs) induced after stimulation with a ligand for CLEC10A. CD1c^+^ DCs were sorted from PBMCs of healthy donors. Subsequently, cells were stimulated with MUC-1-(Tn)_2_ (triangles) or non-glycosylated MUC-1 (squares) as control together with (red curves) or without 5 µg/ml R848 (black curves) for different time points (0, 5, 15, 30, and 60 min) at 37°C. Afterward, the cells were fixed and permeabilized using BD Perm Buffer III and intracellularly stained for phosphorylated **(A)** NFκB p65, **(B)** p38 MAPK, **(C)** IKKγ, **(D)**, JNK, **(E)** ERK-1/2, and **(F)** CREB/ATF-1 with BD Phosflow antibodies. Diagrams show ΔMFI ± SD for two donors. ΔMFI = MFI_antibody_ − MFI_Isotype_.

Since the combination of MUC-1-(Tn)_2_ and R848 enhanced the secretion of cytokines by CD1c^+^ DCs, we wondered, if key signaling pathways were influenced in CD1c^+^ DCs. We therefore incubated the cells with the glycosylated MUC-1-(Tn)_2_ or the non-glycosylated MUC-1 in the presence or absence of the TLR ligand R848 as described earlier. We performed intracellular FACS staining for the detection of phosphorylation in signaling cascade proteins and of transcription factors, such as NFκB p65, p38 MAPK, IKKγ, JNK, ERK-1/2, or CREB/ATF-1. Although all of the tested proteins are important components in intracellular signal transduction, we could not detect any differences in their phosphorylation states after costimulation of CD1c^+^ with MUC-1-(Tn)_2_ and R848 (Figure 7).

## Discussion

Here, we investigated the expression of CLEC10A in various immune cell populations and found a selective presence of this CLR on CD1c^+^ DCs. Our data indicate that CLEC10A is rapidly internalized upon antibody binding. Although MUC-1-(Tn)_2_, a natural ligand for CLEC10A, specifically binds to this DC subset, signaling events were not directly induced. However, we detected a slightly enhanced secretion of selective cytokines induced by the TLR7/8 ligand R848 upon coadministration of MUC-1-(Tn)_2_.

CLEC10A belongs to the family of CLRs. CLRs are responsible for the recognition of sugar structures on bacteria and cancer cells (40, 50–52). For CLEC10A, the specific ligand N-acetylgalactosamine was recently described (41, 44). Although CLEC10A was originally identified on human moDCs (38), we previously found a high mRNA expression in human CD1c^+^ DCs isolated from blood, spleen, and thymus (Figure 1) (15), which is in accordance to other published transcriptomic data (16, 17, 36). This led us to the investigation of CLEC10A protein levels on immune cell populations. Thereby, we found a selective CLEC10A protein expression on human CD1c^+^ DCs. While protein expression of CLEC10A on a human subpopulation of blood CD1c^+^ DCs was already reported by Lundberg et al. (53, 54), we here show that CLEC10A is expressed on the majority of blood as well as lymphoid tissue CD1c^+^ DCs. In contrast to Lundberg et al., we demonstrate that CLEC10A is specifically expressed on CD1c^+^ DCs, but not on CD141^+^ DCs. Our data are further supported by the transcriptional profiling of human lymphohematopoietic DC subpopulations [Figure 1; (15)] as well as our ligand binding assays using the CLEC10A ligand MUC-1-(Tn)_2_ on various immune cell populations (Figure 5). Although we and others found a CLEC10A mRNA expression in monocytes in lymphohematopoietic tissues, we could detect no or a very moderate protein expression (Figure 2) (37). The same was true for CD141^+^ DCs and pDCs (Figures 1 and 2). We here suggest that the lectin receptor CLEC10A might be a useful marker for a more delicate gating of CD1c^+^ DCs, resulting in a better separation from monocyte-related cells.

In dependency on the antibody clone used, recent studies showed different results on the expression of CLEC10A on immune cells. Wong et al. compared the transcriptome of classical, non-classical, and intermediate monocytes and found a specific expression of the mRNA in intermediate monocytes compared to the other two populations of monocytes (37). However, the protein expression of CLEC10A on intermediate monocytes was only marginal and neither the mRNA nor the protein expression was compared to other cell populations of the immune system (37). In our experiments, we identified a small population of CLEC10A^+^ monocytes in the blood (Figure 2), which might represent the described intermediate monocytes of Wong et al (37). In another study, Li et al. generated an antibody against CLEC10A (here named DC-ASGPR) (55). In contrast to our obtained data, the anti-DC-ASGPR antibody (clone 49C11) bound to all myeloid DCs (HLA-DR^+^ CD11c^+^ CD123^−^), CD14^+^ monocytes, and CD19^+^ B cells (55), while we could not observe binding to blood B cells and the majority of monocytes (Figure 2). Moreover, our data reveal that only CD1c^+^ DCs were positive for CLEC10A, while other CD11c^+^ DCs such as CD16^+^ DCs and CD141^+^ DCs were represented among the CLEC10A^−^ DCs (Figure 1). As Valladeau et al. reported that short and long isoforms of CLEC10A (DC-ASGPR) exist (56), we here speculate that the antibody used in our study (clone: H037G3) and the antibody used by Li et al. (49C11) might recognize different isoforms of CLEC10A, which could explain the observed expression differences. The system of different CLEC10A isoforms expressed on either DCs or monocytes/macrophages would also reflect a high similarity to the murine system with CD301a and CD301b expressed on macrophages and DCs, respectively (41, 57). In summary, as the natural ligand of CLEC10A (44) demonstrated a similar binding behavior as the CLEC10A antibody (Figure 5) and as our and other’s transcriptomic analyses (Figure 1) revealed a strong specificity of CLEC10A for CD1c^+^ DCs, the receptor CLEC10A is approved to be a good marker for CD1c^+^ DCs.

Interestingly, data of Breton et al. indicate that, in addition to fully differentiated CD1c^+^ DCs, CD1c^+^ DC-committed pre-cDCs also selectively expressed the mRNA of CLEC10A (17). In contrast, non-committed as well as CD141^+^ DC-committed pre-cDCs lacked the mRNA expression for CLEC10A (17). Although the protein expression has to be confirmed, these data suggest the usage of CLEC10A as a selective lineage marker in the development of CD1c^+^ DCs (Figure 1).

As most *in vivo* studies on immunological questions are performed in the murine system, it is also of major importance to identify the most similar antigen-presenting cells in mice in man. For the CD8^+^/CD103^+^ subset in mice and the CD141^+^ DCs as their potential human counterpart, XCR1 and CLEC9A have been suggested as universal subset-defining markers for DCs, which potentially excel in cross-presentation of engulfed antigens (33, 34, 58–61). However, there is no such marker described for murine CD11b^+^ DCs and human CD1c^+^ DCs, which is not also expressed by monocytic lineages (such as CD172a, also known as SIRPα). In mice, two homologs of CLEC10A exist, named MGL1 (CD301a) and MGL2 (CD301b), respectively. These two CLRs exhibit different expression patterns as MGL1 was found on macrophages in the skin, whereas MGL2 was expressed on dermal CD11b^+^ DCs (57). Human CLEC10A is more closely related to murine MGL2 as both can recognize similar ligands (40, 44). Although there are controversial reports on the protein expression of MGL2 on murine splenic CD11b^+^ DCs (16, 41, 51), we here propose that CLEC10A might not only be a suitable lineage but also a cross-species marker.

In addition to the selective expression of CLEC10A on CD1c^+^ DCs, we further demonstrated that CLEC10A internalizes rapidly into CD1c^+^ DCs upon binding of a specific antibody (Figure 3). A similar behavior has been reported for DEC205 (9, 62, 63). The rapid uptake is a prerequisite for the usage of CLEC10A as a potential antigen-targeting receptor. Antigen targeting takes advantage of fusing a receptor-specific antibody with an antigen of choice, such as viral, bacterial, or cancer antigens. This technique has successfully been used for the induction of immune responses against various antigens (9, 19–22, 24, 28, 64). However, till date there was no suitable CD1c^+^ DC-specific targeting receptor described. We here propose CLEC10A as a potential antigen-targeting receptor for human CD1c^+^ DCs.

In future studies, it will be important to analyze the compartmentalization of CLEC10A by the bound antigen-targeting antibody or the interacting natural ligand. Other lectin receptors such as the murine and human DEC205 receptors localize to late endosomes after uptake into the cell (63, 65). As the efficiency of crosspresentation in human DCs is partially dependent on the antigen routing to late or early endosomes, it will be important to follow the internalization of the CLEC10A receptor, its ligands, or its respective antigen-targeting antibodies (65–67). Importantly, we found no intracellular activation upon binding of the anti-CLEC10A antibody to its receptor (data not shown), supporting our previous findings that internalization and signaling might be separate events (28). As CLEC10A was recently demonstrated to leap into HLA I and II rich compartments in human immature moDCs (68), we propose that targeting of CLEC10A using antigen-targeting antibodies will lead to a presentation of antigens, both on HLA I and II.

Moreover, we investigated the influence of a natural ligand of CLEC10A, MUC-1-(Tn)_2_, on CD1c^+^ DCs. As expected, MUC-1-(Tn)_2_ bound solely to CD1c^+^ DCs, but not to other immune cells (Figure 5). In our analyses, we have not observed a stimulatory capacity of the ligand alone on CD1c^+^ DCs. However, an additional costimulation of sorted CD1c^+^ DCs with a ligand for CLEC10A and the TLR7/8 ligand R848 induced a slightly increased secretion of the cytokines IL-8, IL-10, and TNFα in comparison to R848 alone (Figure 6). This is in line with a study by van Vliet et al., which also showed an enhanced secretion of IL-10 and TNFα after stimulation of moDCs with TLR2 ligands in combination with either anti-CLEC10A antibodies or CLEC10A ligands (49). Furthermore, Li et al. showed that stimulation of IFN-DCs, which were generated from blood monocytes using GM-CSF and IFNα, with an anti-CLEC10A antibody induced ERK and p38 phosphorylation and secretion of IL-10 (55). In contrast to the study by van Vliet et al., an additional TLR stimulation was not necessary. This might be due to the culture with IFNα, which led to a sufficient activation of moDCs (49, 55). In contrast to these studies, we could not observe any additional phosphorylation of ERK in CD1c^+^ DCs upon stimulation with ligands for CLEC10A by Phosflow analyses with or without TLR stimulation (Figure 7) (49, 55).

As the TLR7/8 ligand R848 alone induces high secretion of IL-10 by CD1c^+^ DCs (Figure 6), the additional stimulation of CLEC10A could lead to only subtle changes in ERK phosphorylation, which we could not detect with Phosflow analysis. Furthermore, the induced signaling by a TLR is influenced by the localization of the TLR (69, 70). Therefore, the different localization of TLR7/8 and TLR2 could lead to the activation of different signaling molecules. Even TLR ligands targeting the same receptor induce different signaling events dependent on the targeted ectodomain of the TLR. Although both R848 and RNA40 activated TLR8, only R848 induced phosphorylation of ERK1 (71). Additionally, the used cell type influences the induced signaling after TLR stimulation, too (72). Since moDCs are more similar to human inflammatory DCs than to CD1c^+^ DCs (73), this might additionally explain the observed difference in TLR signaling.

Stimulation of CD1c^+^ DCs with the TLR3 ligand pIC together with glycosylated MUC1-(Tn)_2_ did not induce enhanced cytokine secretion, although the CD1c^+^ DCs showed a high expression of costimulatory molecules (Figure 6). Overall, the detected cytokine levels were notably lower compared to stimulation with the TLR7/8 ligand R848. This is in accordance with published data by other groups, who also showed a differential cytokine pattern induced by TLR3 and TLR7/8 stimulation (47, 48, 74). While TLR7/8 stimulation induces inflammatory cytokines, such as IL-6, IL-12, and TNFα, stimulation with pIC results mainly in secretion of IFNλ as well as chemokines, such as RANTES and IP-10 (47, 48, 74). As TLR3, in contrast to TLR7/8, signals independent of MyD88 *via* TRIF and IRF3, we assume that this difference in cytokine secretion is attributed to the differential signaling pathways that are induced by TLR3 and TLR7/8 (75).

Overall, CLEC10A showed a specific expression on CD1c^+^ DCs in different lymphohematopoietic tissues rendering it as a unique marker for this subset to clearly separate it from monocytic lineages. CLEC10A did not only bind its natural ligand but also internalized rapidly upon binding of a specific antibody. Furthermore, ligands for CLEC10A alone did not induce activation or cytokine secretion by CD1c^+^ DCs. This is a favorable feature for future immunotherapeutic approaches, since thereby antigen targeting of CLEC10A could not only be used to induce immunity but also tolerance under non-stimulatory conditions (22, 76–78).

## Ethics Statement

This study was carried out in accordance with the recommendations of the local ethical committee (Ethikkommission der Friedrich-Alexander-Universität Erlangen-Nürnberg) with written informed consent from all subjects. All subjects gave written informed consent in accordance with the Declaration of Helsinki. The protocol was approved by the local ethical committee (Ethikkommission der Friedrich-Alexander-Universität Erlangen-Nürnberg).

## Author Contributions

LH performed the experiments with participation by GFH, SB, and LHa; and JJL, FG-M, and S-IN provided MGL ligands. GH provided microarray data. CL analyzed microarray data. AH, AP, and RC ensured human tissue sample supply. GH and CL contributed to the review of the manuscript. LH, FN, and DD contributed to the data analysis and interpretation as well as discussions. LH and DD designed the study; and LH, CL, and DD wrote and revised the manuscript.

## Conflict of Interest Statement

The authors declare that the research was conducted in the absence of any commercial or financial relationships that could be construed as a potential conflict of interest.

## References

[1] Banchereau J Steinman RM. Dendritic cells and the control of immunity. Nature (1998) 392:245–52. 10.1038/32588 9521319

[2] Medzhitov R Janeway CJ. Innate immune recognition: mechanisms and pathways. Immunol Rev (2000) 173:89–97. 10.1034/j.1600-065X.2000.917309.x 10719670

[3] Villadangos JA Young L. Antigen-presentation properties of plasmacytoid dendritic cells. Immunity (2008) 29:352–61. 10.1016/j.immuni.2008.09.002 18799143

[4] Swiecki M Colonna M. The multifaceted biology of plasmacytoid dendritic cells. Nat Rev Immunol (2015) 15:471–85. 10.1038/nri3865 26160613 PMC4808588

[5] Reizis B Bunin A Ghosh HS Lewis KL Sisirak V. Plasmacytoid dendritic cells: recent progress and open questions. Annu Rev Immunol (2011) 29:163–83. 10.1146/annurev-immunol-031210-101345 21219184 PMC4160806

[6] Merad M Sathe P Helft J Miller J Mortha A. The dendritic cell lineage: ontogeny and function of dendritic cells and their subsets in the steady state and the inflamed setting. Annu Rev Immunol (2013) 31:563–604. 10.1146/annurev-immunol-020711-074950 23516985 PMC3853342

[7] Guilliams M Ginhoux F Jakubzick C Naik SH Onai N Schraml BU Dendritic cells, monocytes and macrophages: a unified nomenclature based on ontogeny. Nat Rev Immunol (2014) 14:571–8. 10.1038/nri3712 25033907 PMC4638219

[8] Heidkamp GF Lehmann CHK Heger L Baransk A Hoffmann A Lühr J Functional specialization of dendritic cell subsets. Encyclopedia of Cell Biology. Elsevier (2016). p. 588–604. 10.1016/B978-0-12-394447-4.30076-1

[9] Dudziak D Kamphorst AO Heidkamp GF Buchholz VR Trumpfheller C Yamazaki S Differential antigen processing by dendritic cell subsets in vivo. Science (2007) 315:107–11. 10.1126/science.1136080 17204652

[10] Hildner K Edelson BT Purtha WE Diamond M Matsushita H Kohyama M Batf3 deficiency reveals a critical role for CD8 + dendritic cells in cytotoxic T cell immunity. Science (2008) 322:1097–100. 10.1126/science.1164206 19008445 PMC2756611

[11] Schlitzer A McGovern N Teo P Zelante T Atarashi K Low D IRF4 transcription factor-dependent CD11b+ dendritic cells in human and mouse control mucosal IL-17 cytokine responses. Immunity (2013) 38:970–83. 10.1016/j.immuni.2013.04.011 23706669 PMC3666057

[12] Persson E Uronen-Hansson H Semmrich M Rivollier A Hägrbrand K Marsal J IRF4 transcription-factor-dependent CD103+CD11b+ dendritic cells drive mucosal T helper 17 cell differentiation. Immunity (2013) 38:958–69. 10.1016/j.immuni.2013.03.009 23664832

[13] Williams JW Tjota MY Clay BS Vander Lugt B Bandukwala HS Hrusch CL Transcription factor IRF4 drives dendritic cells to promote Th2 differentiation. Nat Commun (2013) 4:1–12. 10.1038/ncomms3990 24356538 PMC4003872

[14] Gao Y Nish S Jiang R Hou L Licona-Limón P Weinstein J Control of T helper 2 responses by transcription factor IRF4-dependent dendritic cells. Immunity (2013) 39:722–32. 10.1016/j.immuni.2013.08.028 24076050 PMC4110745

[15] Heidkamp GF Sander J Lehmann CHK Heger L Eissing N Baranska A Human lymphoid organ dendritic cell identity is predominantly dictated by ontogeny, not tissue microenvironment. Sci Immunol (2016) 1:40–50. 10.1126/sciimmunol.aai7677 28783692

[16] Watchmaker PB Lahl K Lee M Baumjohann D Morton J Kim SJ Comparative transcriptional and functional profiling defines conserved programs of intestinal DC differentiation in humans and mice. Nat Immunol (2014) 15:98–108. 10.1038/ni.2768 24292363 PMC3942165

[17] Breton G Zheng S Valieris R Tojal da Silva I Satija R Nussenzweig MC. Human dendritic cells (DCs) are derived from distinct circulating precursors that are precommitted to become CD1c + or CD141 + DCs. J Exp Med (2016) 213:2861–70. 10.1084/jem.20161135 27864467 PMC5154947

[18] Guilliams M Dutertre CA Scott CL McGovern N Sichien D Chakarov S Unsupervised high-dimensional analysis aligns dendritic cells across tissues and species. Immunity (2016) 45:669–84. 10.1016/j.immuni.2016.08.015 27637149 PMC5040826

[19] Lehmann C Heger L Heidkamp G Baranska A Lühr J Hoffmann A Direct delivery of antigens to dendritic cells via antibodies specific for endocytic receptors as a promising strategy for future therapies. Vaccines (Basel) (2016) 4:8. 10.3390/vaccines4020008 27043640 PMC4931625

[20] Neubert K Lehmann CHK Heger L Baranska A Staedtler AM Buchholz VR Antigen delivery to CD11c+CD8− dendritic cells induces protective immune responses against experimental melanoma in mice in vivo. J Immunol (2014) 192:5830–8. 10.4049/jimmunol.1300975 24829411

[21] Sancho D Mourão-Sá D Joffre OP Schulz O Rogers NC Pennington DJ Tumor therapy in mice via antigen targeting to a novel, DC-restricted C-type lectin. J Clin Invest (2008) 118:2098–110. 10.1172/JCI34584 18497879 PMC2391066

[22] Joffre OP Sancho D Zelenay S Keller AM Reis e Sousa C. Efficient and versatile manipulation of the peripheral CD4+ T-cell compartment by antigen targeting to DNGR-1/CLEC9A. Eur J Immunol (2010) 40:1255–65. 10.1002/eji.201040419 20333625 PMC3064981

[23] Bonifaz LC Bonnyay DP Charalambous A Darguste DI Fujii S-I Soares H In vivo targeting of antigens to maturing dendritic cells via the DEC-205 receptor improves T cell vaccination. J Exp Med (2004) 199:815–24. 10.1084/jem.20032220 15024047 PMC2212731

[24] Wang B Zaidi N He L-Z Zhang L Kuroiwa JM Keler T Targeting of the non-mutated tumor antigen HER2/neu to mature dendritic cells induces an integrated immune response that protects against breast cancer in mice. Breast Cancer Res (2012) 14:R39. 10.1186/bcr3135 22397502 PMC3446373

[25] Macho-Fernandez E Cruz LJ Ghinnagow R Fontaine J Bialecki E Frisch B Targeted delivery of α-galactosylceramide to CD8α ^+^ dendritic cells optimizes type I NKT cell–based antitumor responses. J Immunol (2014) 193:961–9. 10.4049/jimmunol.1303029 24913977

[26] Hawiger D Inaba K Dorsett Y Guo M Mahnke K Rivera M Dendritic cells induce peripheral T cell unresponsiveness under steady state conditions in vivo. J Exp Med (2001) 194:769–79. 10.1084/jem.194.6.769 11560993 PMC2195961

[27] Do Y Koh H Park CG Dudziak D Seo P Mehandru S Targeting of LcrV virulence protein from *Yersinia pestis* to dendritic cells protects mice against pneumonic plague. Eur J Immunol (2010) 40:2791–6. 10.1002/eji.201040511 20812236

[28] Lehmann CHK Baranska A Heidkamp GF Heger L Neubert K Lühr JJ DC subset-specific induction of T cell responses upon antigen uptake via Fcγ receptors in vivo. J Exp Med (2017) 214:jem.20160951 10.1084/jem.20160951 PMC5413326 28389502

[29] Heidkamp GF Neubert K Haertel E Nimmerjahn F Nussenzweig MC Dudziak D. Efficient generation of a monoclonal antibody against the human C-type lectin receptor DCIR by targeting murine dendritic cells. Immunol Lett (2010) 132:69–78. 10.1016/j.imlet.2010.06.002 20566350 PMC2997960

[30] Pack M Trumpfheller C Thomas D Park CG Granelli-Piperno A Münz C DEC-205/CD205+ dendritic cells are abundant in the white pulp of the human spleen, including the border region between the red and white pulp. Immunology (2008) 123:438–46. 10.1111/j.1365-2567.2007.02710.x 17944899 PMC2433334

[31] Meyer-Wentrup F Benitez-Ribas D Tacken PJ Punt CJA Figdor CG de Vries IJM Targeting DCIR on human plasmacytoid dendritic cells results in antigen presentation and inhibits IFN-α production. Blood (2008) 111:4245–53. 10.1182/blood-2007-03-081398.The 18258799

[32] Sancho D Joffre OP Keller AM Rogers NC Martínez D Hernanz-Falcón P Identification of a dendritic cell receptor that couples sensing of necrosis to immunity. Nature (2009) 458:899–903. 10.1038/nature07750 19219027 PMC2671489

[33] Poulin LF Salio M Griessinger E Anjos-Afonso F Craciun L Chen J-L Characterization of human DNGR-1+ BDCA3+ leukocytes as putative equivalents of mouse CD8alpha+ dendritic cells. J Exp Med (2010) 207:1261–71. 10.1084/jem.20092618 20479117 PMC2882845

[34] Poulin LF Reyal Y Uronen-Hansson H Schraml B Sancho D Murphy KM DNGR-1 is a specific and universal marker of mouse and human Batf3-dependent dendritic cells in lymphoid and non-lymphoid tissues. Blood (2012) 119:6052–62. 10.1182/blood-2012-01-406967 22442345

[35] Caminschi I Proietto AI Ahmet F Kitsoulis S Shin Teh J Lo JCY The dendritic cell subtype-restricted C-type lectin Clec9A is a target for vaccine enhancement. Blood (2008) 112:3264–73. 10.1182/blood-2008-05-155176 18669894 PMC2569177

[36] Villani A-C Satija R Reynolds G Sarkizova S Shekhar K Fletcher J Single-cell RNA-seq reveals new types of human blood dendritic cells, monocytes, and progenitors. Science (2017) 356:eaah4573. 10.1126/science.aah4573 28428369 PMC5775029

[37] Wong KL Tai JJ-Y Wong W-C Han H Sem X Yeap W-H Gene expression profiling reveals the defining features of the classical, intermediate, and nonclassical human monocyte subsets. Blood (2011) 118:e16–31. 10.1182/blood-2010-12-326355 21653326

[38] Higashi N Fujioka K Denda-Nagai K Hashimoto S Nagai S Sato T The macrophage C-type lectin specific for galactose/N-acetylgalactosamine is an endocytic receptor expressed on monocyte-derived immature dendritic cells. J Biol Chem (2002) 277:20686–93. 10.1074/jbc.M202104200 11919201

[39] Tsuiji M Fujimori M Ohashi Y Higashi N Onami TM Hedrick SM Molecular cloning and characterization of a novel mouse macrophage C-type lectin, mMGL2, which has a distinct carbohydrate specificity from mMGL1. J Biol Chem (2002) 277:28892–901. 10.1074/jbc.M203774200 12016228

[40] Singh SK Streng-Ouwehand I Litjens M Weelij DR García-Vallejo JJ van Vliet SJ Characterization of murine MGL1 and MGL2 C-type lectins: distinct glycan specificities and tumor binding properties. Mol Immunol (2009) 46:1240–9. 10.1016/j.molimm.2008.11.021 19162326

[41] Denda-Nagai K Aida S Saba K Suzuki K Moriyama S Oo-puthinan S Distribution and function of macrophage galactose-type C-type lectin 2 (MGL2/CD301b). J Biol Chem (2010) 285:19193–204. 10.1074/jbc.M110.113613 20304916 PMC2885198

[42] Core Team R R: A Language and Environment for Statistical Computing. (2016). Available from: https://www.r-project.org/ (Accessed: December 19, 2017).

[43] Warnes GR Bolker B Bonebakker L Gentleman R Huber W Liaw A gplots: Various R Programming Tools for Plotting Data. R Package Version 3.0.1. (2016). Available from: https://cran.r-project.org/package=gplots/ (Accessed: December 19, 2017).

[44] Artigas G Monteiro JT Hinou H Nishimura S-I Lepenies B Garcia-Martin F. Glycopeptides as targets for dendritic cells: exploring MUC1 glycopeptides binding profile toward macrophage galactose-type lectin (MGL) orthologs. J Med Chem (2017) 60:9012–21. 10.1021/acs.jmedchem.7b01242 29045792

[45] Fossum E Grodeland G Terhorst D Tveita AA Vikse E Mjaaland S Vaccine molecules targeting Xcr1 on cross-presenting DCs induce protective CD8+ T-cell responses against influenza virus. Eur J Immunol (2015) 45:624–35. 10.1002/eji.201445080 25410055

[46] Gudjonsson A Lysen A Balan S Sundvold-Gjerstad V Arnold-Schrauf C Richter L Targeting influenza virus hemagglutinin to Xcr1+ dendritic cells in the absence of receptor-mediated endocytosis enhances protective antibody responses. J Immunol (2017) 198:2785–95. 10.4049/jimmunol.1601881 28228559

[47] Hémont C Neel A Heslan M Braudeau C Josien R. Human blood mDC subsets exhibit distinct TLR repertoire and responsiveness. J Leukoc Biol (2013) 93:1–11. 10.1189/jlb.0912452 23341538

[48] Nizzoli G Krietsch J Weick A Steinfelder S Facciotti F Gruarin P Human CD1c+ dendritic cells secrete high levels of IL-12 and potently prime cytotoxic T-cell responses. Blood (2013) 122:932–42. 10.1182/blood-2013-04-495424 23794066

[49] van Vliet SJ Bay S Vuist IM Kalay H Garcia-Vallejo JJ Leclerc C MGL signaling augments TLR2-mediated responses for enhanced IL-10 and TNF- secretion. J Leukoc Biol (2013) 94:315–23. 10.1189/jlb.1012520 23744646

[50] Saeland E van Vliet SJ Bäström M van den Berg VCM Geijtenbeek TBH Meijer GA The C-type lectin MGL expressed by dendritic cells detects glycan changes on MUC1 in colon carcinoma. Cancer Immunol Immunother (2007) 56:1225–36. 10.1007/s00262-006-0274-z 17195076 PMC11031027

[51] Singh SK Streng-Ouwehand I Litjens M Kalay H Saeland E van Kooyk Y. Tumour-associated glycan modifications of antigen enhance MGL2 dependent uptake and MHC class I restricted CD8 T cell responses. Int J Cancer (2011) 128:1371–83. 10.1002/ijc.25458 20473945

[52] Hoving JC Wilson GJ Brown GD. Signalling C-Type lectin receptors, microbial recognition and immunity. Cell Microbiol (2014) 16:185–94. 10.1111/cmi.12249 24330199 PMC4016756

[53] Lundberg K Rydnert F Greiff L Lindstedt M. Human blood dendritic cell subsets exhibit discriminative pattern recognition receptor profiles. Immunology (2014) 142:279–88. 10.1111/imm.12252 24444310 PMC4008235

[54] Lundberg K Rydnert F Broos S Andersson M Greiff L Lindstedt M. Allergen-specific immunotherapy alters the frequency, as well as the FcR and CLR expression profiles of human dendritic cell subsets. PLoS One (2016) 11:e0148838. 10.1371/journal.pone.0148838 26863539 PMC4749279

[55] Li D Romain G Flamar A-L Duluc D Dullaers M Li X-H Targeting self- and foreign antigens to dendritic cells via DC-ASGPR generates IL-10–producing suppressive CD4 ^+^ T cells. J Exp Med (2012) 209:109–21. 10.1084/jem.20110399 22213806 PMC3260876

[56] Valladeau J Duvert-Frances V Pin J-J Kleijmeer MJ Ait-Yahia S Ravel O Immature human dendritic cells express asialoglycoprotein receptor isoforms for efficient receptor-mediated endocytosis. J Immunol (2001) 167:5767–74. 10.4049/jimmunol.167.10.5767 11698450

[57] Kumamoto Y Denda-Nagai K Aida S Higashi N Irimura T MGL2+ dermal dendritic cells are sufficient to initiate contact hypersensitivity in vivo. PLoS One (2009) 4:e5619 10.1371/journal.pone.0005619 19440334 PMC2680031

[58] Bachem A Güttler S Hartung E Ebstein F Schaefer M Tannert A Superior antigen cross-presentation and XCR1 expression define human CD11c+CD141+ cells as homologues of mouse CD8+ dendritic cells. J Exp Med (2010) 207:1273–81. 10.1084/jem.20100348 20479115 PMC2882837

[59] Bachem A Hartung E Güttler S Mora A Zhou X Hegemann A Expression of XCR1 characterizes the Batf3-dependent lineage of dendritic cells capable of antigen cross-presentation. Front Immunol (2012) 3:214. 10.3389/fimmu.2012.00214 22826713 PMC3399095

[60] Crozat K Guiton R Contreras V Feuillet V Dutertre C-A Ventre E The XC chemokine receptor 1 is a conserved selective marker of mammalian cells homologous to mouse CD8alpha+ dendritic cells. J Exp Med (2010) 207:1283–92. 10.1084/jem.20100223 20479118 PMC2882835

[61] Schreibelt G Klinkenberg LJJ Cruz LJ Tacken PJ Tel J Kreutz M The C-type lectin receptor CLEC9A mediates antigen uptake and (cross-)presentation by human blood BDCA3+ myeloid dendritic cells. Blood (2012) 119:2284–92. 10.1182/blood-2011-08-373944 22234694

[62] Reuter A Panozza SE Macri C Dumont C Li J Liu H Criteria for dendritic cell receptor selection for efficient antibody-targeted vaccination. J Immunol (2015) 194:2696–705. 10.4049/jimmunol.1402535 25653426

[63] Mahnke K Guo M Lee S Sepulveda H Swain SL Nussenzweig M The dendritic cell receptor for endocytosis, DEC-205, can recycle and enhance antigen presentation via major histocompatibility complex class II – positive lysosomal compartments. J Cell Biol (2000) 151:673–83. 10.1083/jcb.151.3.673 11062267 PMC2185598

[64] Matos I Mizenina O Lubkin A Steinman RM Idoyaga J. Targeting *Leishmania* major Antigens to dendritic cells in vivo induces protective immunity. PLoS One (2013) 8:e67453. 10.1371/journal.pone.0067453 23840706 PMC3694010

[65] Cohn L Chatterjee B Esselborn F Smed-Sörensen A Nakamura N Chalouni C Antigen delivery to early endosomes eliminates the superiority of human blood BDCA3 ^+^ dendritic cells at cross presentation. J Exp Med (2013) 210:1049–63. 10.1084/jem.20121251 23569326 PMC3646496

[66] Chatterjee B Smed-Sörensen A Cohn L Chalouni C Vandlen R Lee B-C Internalization and endosomal degradation of receptor-bound antigens regulate the efficiency of cross presentation by human dendritic cells. Blood (2012) 120(10):2011–20. 10.1182/blood-2012-01-402370 22791285

[67] Joffre OP Segura E Savina A Amigorena S. Cross-presentation by dendritic cells. Nat Rev Immunol (2012) 12:557–69. 10.1038/nri3254 22790179

[68] Napoletano C Rughetti A Agervig Tarp MP Coleman J Bennett EP Picco G Tumor-associated Tn-MUC1 glycoform is internalized through the macrophage galactose-type C-type lectin and delivered to the HLA class I and II compartments in dendritic cells. Cancer Res (2007) 67:8358–67. 10.1158/0008-5472.CAN-07-1035 17804752

[69] Kagan JC Su T Horng T Chow A Akira S Medzhitov R TRAM couples endocytosis of toll-like receptor 4 to the induction of interferon-β. Nat Immunol (2008) 9:361–8. 10.1038/ni1569 18297073 PMC4112825

[70] Husebye H Halaas Ø Stenmark H Tunheim G Sandanger Ø Bogen B Endocytic pathways regulate toll-like receptor 4 signaling and link innate and adaptive immunity. EMBO J (2006) 25:683–92. 10.1038/sj.emboj.7600991 16467847 PMC1383569

[71] Colak E Leslie A Zausmer K Khatamzas E Kubarenko AV Pichulik T RNA and imidazoquinolines are sensed by distinct TLR7/8 ectodomain sites resulting in functionally disparate signaling events. J Immunol (2014) 192:5963–73. 10.4049/jimmunol.1303058 24813206 PMC4066583

[72] Sun J Li N Oh KS Dutta B Vayttaden SJ Lin B Comprehensive RNAi-based screening of human and mouse TLR pathways identifies species-specific preferences in signaling protein use. Sci Signal (2016) 9:1–38. 10.1126/scisignal.aab2191 26732763 PMC5381726

[73] Segura E Touzot M Bohineust A Cappuccio A Chiocchia G Hosmalin A Human inflammatory dendritic cells induce Th17 cell differentiation. Immunity (2013) 38:336–48. 10.1016/j.immuni.2012.10.018 23352235

[74] Lundberg AM Drexler SK Monaco C Williams LM Sacre SM Feldmann M Key differences in TLR3/poly I:C signaling and cytokine induction by human primary cells: a phenomenon absent from murine cell systems. Blood (2007) 110:3245–52. 10.1182/blood-2007-02-072934 17660379

[75] Kawai T Akira S. Toll-like receptors and their crosstalk with other innate receptors in infection and immunity. Immunity (2011) 34:637–50. 10.1016/j.immuni.2011.05.006 21616434

[76] Ring S Maas M Nettelbeck DM Enk AH Mahnke K Targeting of autoantigens to DEC205+ dendritic cells in vivo suppresses experimental allergic encephalomyelitis in mice. J Immunol (2013) 191:2938–47. 10.4049/jimmunol.1202592 23945139

[77] Stern JNH Keskin DB Kato Z Waldner H Schallenberg S Anderson A Promoting tolerance to proteolipid protein-induced experimental autoimmune encephalomyelitis through targeting dendritic cells. Proc Natl Acad Sci U S A (2010) 107:17280–5. 10.1073/pnas.1010263107 20855626 PMC2951396

[78] Spiering R Margry B Keijzer C Petzold C Hoek A Wagenaar-Hilbers J DEC205+ dendritic cell-targeted tolerogenic vaccination promotes immune tolerance in experimental autoimmune arthritis. J Immunol (2015) 194:4804–13. 10.4049/jimmunol.1400986 25862815

